# Supplementary material for: An application of node and edge nonlinear hypergraph centrality to a protein complex hypernetwork
Source: PLoS One. 2024 Oct 3;19(10):e0311433. doi: 10.1371/journal.pone.0311433 (PMC11449304; doi:10.1371/journal.pone.0311433)
Supplement: Attachment — Submitted filename: Response to Reviewers.pdf [file pone.0311433.s001.pdf]

Ms Irene Señdina-Nadal
Academic Editor
PloS One
1265 Battery Street, Suite 200
San Francisco, CA 94111
United States

Sarah Lawson
School of Mathematics and Physics
Physics Annexe (6), Level 2
The University of Queensland, St Lucia,
Brisbane, QLD 4072, Australia
0431 947 462
Sarah.lawson@uq.edu.au

23rd August 2024

Dear Ms Señdina-Nadal,

Thank you for considering our manuscript and thank you to the reviewers for their feedback. The points raised have been addressed as follows:

- Please remove any funding-related text from the manuscript and let us know how you would like to update your Funding Statement. Currently, your Funding Statement reads as follows:
  [This work was supported by The Australian Research Council, through the Centre of Excellence for Plant Success
  in Nature and Agriculture (CE200100015) https://www.plantsuccess.org/. Author DD is CI in the CoE. The CoE and ARC had no direct role in study design, data collection and analysis, decision to publish, or preparation of the manuscript.]
  - Funding information has been removed from the acknowledgements. The funding statement is correct.

- We noted in your submission details that a portion of your manuscript may have been presented or published elsewhere. [Paper on arXiv: https://arxiv.org/abs/2406.01880 preprint server only] Please clarify whether this conference proceeding was peer-reviewed and formally published.
  - The submission to arXiv was not peer-reviewed or formally published.

**Response to Reviewers:**

Reviewer #1:

1. The role of zero as a value for $a,b$ deserves some additional explanation, especially when a comparison with the three examples given by Tudisco-Higham is made.
   - Further clarification has been added to manuscript lines 213-214 (revised manuscript with track changes lines 230-231) for b=d=0 and calculations and explanation included in Table 1 for P:(0,b,0,d) (i.e. a=c=0).

2. The authors observe that the rankings obtained don't vary when the variables $a,b,c,d$ belong to the same family, but I miss some additional justification of why this is so in the examples given.
   - Clarification has been added to manuscript lines 228-230 (revised manuscript with track changes lines 248-250) with calculations and explanations included in Table 1.

3. A natural question arises about the vector structure in $\mathbb{R}^4$ of those families above. Perhaps some comment or hint would be welcome in this direction but I leave it to the authors' choice.

- This is solely notation. Explanation has been rewritten in manuscript line 212 (revised manuscript with track changes line 229).

Reviewer #2:

1. I think it would make it easier to digest if there were a summary table of the different function configurations and power configurations and what they correspond to.
    - Table 1 has been added. (Please note that the recommended latexdiff used to track changes causes an issue with this table formatting in the 'revised manuscript with track changes' document however the table is correctly formatted in the 'manuscript' document).

2. It would be nice to compare your findings to a comparable graph approach. Does the state of the art graph measure applied to PINs have even higher scores? … does NSVC do better at identifying essential proteins?
    - Discussion regarding the issues involved in identifying a state-of-the-art graph measure applied to a PIN dataset and comparing this with the NSVC model applied to a PCH dataset have been included in the "Comparison with a classification method applied to a PIN" section beginning on manuscript line 399 (revised manuscript with track changes line 456). We feel that these issues make a sound comparison infeasible. However, if the reviewer still feels that it is critical for publication we have provided a comparison using the MIPS PIN-PCH intersection beginning on manuscript line 419 (revised manuscript with track changes line 476).
    - Should the reviewer agree that the issues with the comparison warrant it infeasible then we propose the comparison is omitted.

3. On page 2 when you define weighted node degree in terms of w(e) and weighted edge degree in terms of v(i) you haven't defined the w and v functions. I'd suggest to avoid v as a weight function since v_i are vertices.
    - Functions defined on manuscript lines 139-141 (revised manuscript with track changes lines 147-150).
    - v(i) replaced by n(i) throughout manuscript.

4. When you introduce the NSVC model please provide a bit more intuition at the front. How should we think of the f, g, phi, psi functions? What are x and y really capturing?
    - Further discussion regarding this has been added to manuscript lines 59-66 (revised manuscript with track changes lines 64-72).

The highlighted changes in the 'Revised Manuscript with Track Changes' document include the changes detailed above, changes required to meet formatting guidelines and changes made to improve readability.

We appreciate your time in reviewing our submission and look forward to your response.

Kind Regards,

Sarah Lawson, The University of Queensland,
School of Mathematics and Physics, Qld 4072, Australia
